# Frauen in Führungspositionen: Chancen und Risiken durch die COVID-19-Pandemie

**DOI:** 10.1007/s10273-021-2900-y

**Published:** 2021-04-17

**Authors:** Vivien-Sophie Gulden, Stephan L. Thomsen

**Affiliations:** Institut für Wirtschaftspolitik, Leibniz Universität Hannover, Königsworther Platz 1, 30167 Hannover, Deutschland

**Keywords:** J13, J16, J71

## Abstract

Die geringe Repräsentation von Frauen in Führungspositionen hat die EU-Kommission dazu veranlasst, Maßnahmen und Ziele für eine gleichberechtigte Führungsverantwortung zu definieren. Zeitgleich haben die Corona-Maßnahmen zu tiefgreifenden Veränderungen der Arbeitsorganisation geführt. Neben einem Überblick zur Situation von Frauen in Führungspositionen wird ein Augenmerk auf die Wirkungen der Corona-Maßnahmen gelegt, um Chancen und Risiken zu identifizieren. Diese Maßnahmen können durch Verhaltensänderungen mit sowohl positiven als auch negativen Effekten verbunden sein. Langfristig dürften Frauen von den veränderten Bedingungen jedoch profitieren.

## Abstract (en)

The low representation of women in leadership positions has induced the EU Commission to define measures and targets for gender equality. Almost simultaneously, the measures adopted in the coronavirus pandemic have led to profound changes in the organisation of work. These can slow or inhibit the process. This article provides an overview of the situation of women in leadership positions and the most important reasons for their underrepresentation. Special attention is paid to the effects of the COVID-19 measures in order to identify opportunities and risks. It becomes clear that the same measures entail both positive and negative effects through behavioural changes. Nevertheless, in the long term, women are likely to benefit significantly from the new conditions.

Anfang 2020 hat die Europäische Kommission die Strategie zur Gleichstellung der Geschlechter 2020 bis 2025 beschlossen und rückt dieses Thema ins Zentrum der europäischen Politik: Frauen soll es möglich werden, in wie für Männer selbstverständlicher Weise berufliche und gesellschaftliche Ziele zu erreichen und ihr Potenzial bestmöglich auszuschöpfen (EU-Kommission, 2020). Neu in dieser Strategie ist insbesondere das dritte Ziel, „Gleichberechtigte Führungsverantwortung in der Gesellschaft", zu dem neben der Forderung auch konkrete Maßnahmen vorgeschlagen werden, um in Zukunft mehr Führungspositionen mit Frauen zu besetzen. Damit bekommt die Gleichstellung, speziell in Bezug auf Führungspositionen, eine hervorgehobene Bedeutung in der politischen Diskussion (Wrohlich, 2020).

Die Diskussion mag im Jahr 2021 überraschen: Man könnte erwarten, dass Frauen und Männer schon gleichermaßen in den höheren und höchsten Stellen vertreten wären. Doch dies ist (noch) nicht der Fall (z. B. AllBright Stiftung, 2020a, 2020b). Auch der Gleichstellungsindex des European Institute for Gender Equality (EIGE) verdeutlicht die klaren Unterschiede auf dem Sektor „Macht" (d. h. Repräsentation in Führungspositionen von Politik, Wirtschaft und Gesellschaft): Mit einem Indexwert von 56,6 (2019) und 59,5 (2020) liegt Deutschland zwar über dem Durchschnitt der Europäischen Union, ist mit Rang 8 in beiden Jahren aber deutlich von der Spitze entfernt (EIGE, 2019, 2020).

Die besondere politische Herausstellung und Förderung folgt dabei maßgeblich der Überlegung, dass Frauen nur dann Vorbilder und Impulsgeberinnen sein können, wenn sie auch als Vorreiterinnen von relevanten Entwicklungen in der Öffentlichkeit wahrgenommen werden. So könne durch die Förderung von Forscherinnen die Akzeptanz weiblicher Karrieren in der Gesellschaft gestärkt werden. Dies würde jüngere Frauen positiv beeinflussen, ebenfalls eine Karriere anzustreben. Sichtbare Rollenvorbilder stärken so das Selbstvertrauen und Selbstbewusstsein nachfolgender Generationen. Dass dies keine Utopien sind, zeigen wissenschaftliche Untersuchungen. So ist nachgewiesen, dass gemischte Führungsteams in Unternehmen zu profitableren Ergebnissen kommen als rein männliche, da sie vielfältiger an Problemen und deren Lösungen arbeiten (vgl. Hunt et al., 2020). Ein größerer Frauenanteil in Führungspositionen kann zudem die geschlechterspezifische Lohnlücke verringern, wie für verschiedene Länder belegt wurde. Die (durchschnittliche) Einkommensangleichung der Geschlechter ist

**Vivien-Sophie Gulden** ist wissenschaftliche Mitarbeiterin am Institut für Wirtschaftspolitik und am Leibniz Forschungszentrum Wissenschaft und Gesellschaft (LCSS) der Leibniz Universität Hannover.

**Prof. Dr. Stephan L. Thomsen** ist Direktor des Instituts für Wirtschaftspolitik und Mitglied im Vorstand des Leibniz Forschungszentrum Wissenschaft und Gesellschaft (LCSS) der Leibniz Universität Hannover.

daher ein Argument einer ausgeglichene(re)n Verteilung von Männern und Frauen in Führungspositionen.

Die Pandemie beleuchtet viele gesellschaftliche Prozesse wie durch ein Brennglas. Sie kann sich dabei sowohl positiv als auch negativ auf eine gleichberechtigte Führungsverantwortung der Geschlechter auswirken; zugleich kann sie helfen, mögliche Schwachstellen der bestehenden Maßnahmen herauszustellen. Denn trotz der bestehenden Maßnahmen und umfangreichen Forschung ist die Frage nach den Gründen für die geringe Realisation der Karrierechancen von Frauen auf dem deutschen Arbeitsmarkt nicht vollständig beantwortet. Weithin bekannte und bestätigte Ursachen umfassen Einschränkungen in der Vereinbarkeit von Familie und Beruf, z. B. durch Inflexibilität der Arbeitsprozesse oder die Orientierung der Arbeitsabläufe an männlichen Rollenmustern. Dazu kommen Verhaltensunterschiede, die sich z.B. in Präferenzen oder der Bereitschaft zum Wettbewerb ausdrücken. Durch die in der Pandemie ergriffenen Maßnahmen ist es nun geradezu sprunghaft zu einer deutlich vergrößerten Sensibilisierung für flexible Arbeitszeitmodelle, für Homeoffice und für Videokommunikation und weniger Geschäftsreisen gekommen. Zugleich wurden geschlechtsunabhängig die Grenzen der Vereinbarkeit von Kinderbetreuung bzw. Sorgearbeit mit dem Beruf deutlich. Auch wenn die langfristigen Folgen noch nicht bekannt sind, hat es eine Akzeptanzverschiebung im Hinblick auf die neue Arbeitsorganisation auf den Führungsebenen gegeben.

Dieser Beitrag gibt einen Überblick zur Situation von Frauen in Führungspositionen sowie den wichtigsten, in der Literatur belegten Ursachen. Ein besonderes Augenmerk wird dabei auf die bereits bestätigten sowie die zu erwartenden Wirkungen der Corona-Maßnahmen auf die Chancen und Risiken für Frauen in Führungspositionen gelegt. Es wird deutlich, dass dieselben Maßnahmen durch Verhaltensänderungen sowohl Chancen als auch Risiken bergen. Dabei dürften Frauen langfristig aber am stärksten von den veränderten Bedingungen profitieren.

## Frauen in Führungspositionen

Bereits infolge der ersten, aber umso mehr der zweiten Bildungsexpansion seit der Jahrtausendwende erreichen inzwischen mehr Frauen als Männer Hochschulzugangsberechtigungen (2019: 53,8 %) und Hochschulabschlüsse (2019: 51,7 %) (Destatis, 2020). Diese Verbesserung der Chancen auf Hochschulbildung und die höhere Präsenz von Frauen an den Universitäten sollte mit einer Verbesserung der Situation auf dem Arbeitsmarkt einhergehen. Dennoch bestehen substanzielle Unterschiede zwischen den Geschlechtern in der anschließenden Karriere, und die überdurchschnittlichen Anteile in der höheren Bildung

Abbildung 1

**Frauenanteile nach Bildungsabschlüssen und in höchsten Führungspositionen**

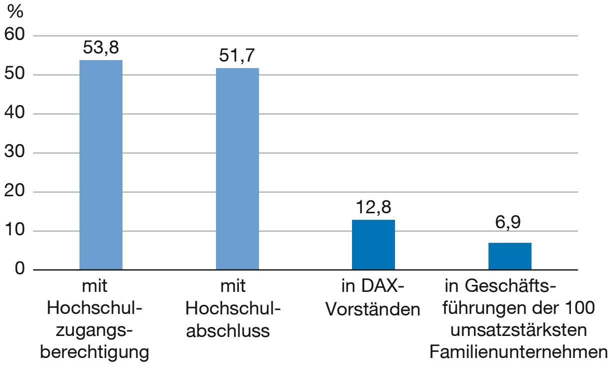

Quellen: Werte zu Hochschulzugangsberechtigung und Hochschulabschlüssen für 2019 (Destatis, 2020); Werte für höchste Führungspositionen für 2020 (AllBright Stiftung, 2020a, 2020b); eigene Darstellung.

spiegeln sich bisher nicht in vergleichbar hohen Frauenanteilen in den Führungsebenen der Unternehmen wider (vgl. Abbildung 1). Zahlen aus den Dax-Vorständen (2020: 12,8 %) oder den Geschäftsführungen der 100 umsatzstärksten Familienunternehmen in Deutschland (6,9 %) dokumentieren die geringen Anteile (vgl. Abbildung 1). Tendenziell ist dabei ein inverser Zusammenhang zur Betriebsgröße in der Privatwirtschaft festzustellen: Während der Frauenanteil in der ersten Führungsebene in Betrieben mit weniger als 49 Beschäftigten 2018 bei rund einem Viertel lag, waren es bei Großbetrieben mit mindestens 500 Beschäftigten nur 14 % (Kohaut und Möller, 2019).

Besondere Beachtung erfahren hier börsennotierte Unternehmen. So verabschiedete die Bundesregierung 2015 ein Gesetz für die gleichberechtigte Teilhabe von Frauen und Männern an Führungspositionen in der Privatwirtschaft und im öffentlichen Dienst (FüPoG, BGBl, Nr. 17 vom 30.4.2015), das börsennotierte Unternehmen mit paritätischer Mitbestimmung zu einer nachweisbaren Frauenquote von 30 % in ihren Aufsichtsräten verpflichtet. Daneben soll(t)en weitere börsennotierte oder mitbestimmte Unternehmen eigene Zielgrößen für den Frauenanteil in Aufsichtsräten, Vorständen und oberen Führungspositionen definieren (Arndt und Wrohlich, 2019). Fünf Jahre nach Verabschiedung des Gesetzes erreichen nur 37 % der DAX-Unternehmen einen Frauenanteil in den Aufsichtsräten von mindestens 40 %. Nur eins der 30 DAX-Unternehmen hat aktuell einen Frauenanteil im Vorstand von 38 % (Telekom), acht werden von einem rein männlichen Vorstand geführt (FidAR, 2020). Deutschland schließt im Vergleich mit den USA, Schweden, Großbritannien, Frankreich und Polen am schlechtesten ab (AllBright Stiftung, 2020b). Diese Beispiele verdeutlichen, dass sich Veränderungen auf freiwilliger Basis, wenn überhaupt, nur sehr langsam vollziehen.

Auch hinsichtlich der Definition der Zielgrößen besteht Spielraum: So haben sich 55 der 160 Unternehmen in DAX30, MDAX und SDAX eine Zielgröße von 0 % für den Frauenanteil in Vorständen und Aufsichtsräten gesetzt. Diese Zielgröße signalisiert Managerinnen innerhalb des Unternehmens, dass ihnen das Vorrücken in den Vorstand nicht zugetraut wird; Managerinnen von außerhalb erwarten, dass keine weibliche Kompetenz gefragt ist (AllBright Stiftung, 2020b). Im aktuellen Gesetzentwurf zur Ergänzung und Änderung der Regelungen für die gleichberechtigte Teilhabe von Frauen an Führungspositionen in der Privatwirtschaft und im öffentlichen Dienst (Zweites Führungspositionengesetz FüPo II) von Januar 2021 wird daher die verpflichtende Festschreibung eines Mindestanteils von Frauen in Vorständen mit mehr als drei Mitgliedern in großen deutschen Unternehmen verbunden mit effektiveren Sanktionen bei unbegründeter Nichtbeachtung gefordert.

## Ursachen und Gründe

Für den geringeren Frauenanteil in Führungspositionen finden sich vielfältige Gründe. So spielen die gesellschaftlichen Rollenmodelle und geschlechterstereotype Zuschreibungen (Adriaans et al., 2020) sowie kulturelle Normen (Adams und Kirchmaier, 2015) eine Rolle. Vielfach nachgewiesen sind Hürden, die es Frauen erschweren, in höhere Positionen (Glass Ceiling Effect) oder aus schlechter bezahlten Jobs (Sticky Floor Effect) aufzusteigen.

Außerdem tragen die steuerlichen Anreize des Ehegattensplittings (Schrenker und Zucco, 2020) genauso wie Einschränkungen in der Kinderbetreuung und daraus resultierend die Einschränkungen in der Vereinbarkeit von Familie und Beruf (Adams und Kirchmaier, 2015) zu einer geringeren Erwerbstätigkeit von Frauen bei. Eine weitere Ursache sind die geläufigen Arbeitszeitmodelle und Erwartungen an Personen in hohen Führungspositionen, die in der Regel an der Lebensrealität von Männern ausgerichtet sind (Wrohlich, 2020); Mutterschaft, Teilzeitarbeit und Unterbrechungen der Karriere sind somit hinderlich (Bertrand, 2010).

Auch die fehlende Sichtbarkeit von Frauen in Führungspositionen wird als Ursache genannt (Kohaut und Möller, 2019). Von mehr erfolgreichen Frauen, die als Vorbilder agieren, wird eine Signalwirkung für jüngere Generationen erwartet. Zudem würden die geschlechterstereotypen Vorstellungen bezüglich der Führungsqualität und -fähigkeit von Frauen zunehmend durchbrochen.[1]

1 Gleichzeitig könnte eine vermehrte Tätigkeit von Männern bei der Kinderbetreuung und im Haushalt die Einstellungen des Umfeldes zu diesen stereotypen Zuschreibungen verändern (Adriaans et al., 2020).

Gut dokumentiert sind außerdem Verhaltensunterschiede, die auf Präferenzen und psychologische Faktoren zurückgeführt werden können. Frauen sind im Durchschnitt eher risikoscheu als Männer. Dadurch streben sie seltener eine Führungsposition an (Bertrand, 2010) und bevorzugen Tätigkeiten, die mit dem Familienleben kompatibel sind (Busch, 2013). Auch haben Frauen im Durchschnitt andere Prioritäten als Männer in Bezug auf Beruf und Karriere sowie Einkommenserwartungen bzw. -ansprüche (Briel et al., 2020). Trotz der bekannten Einkommensunterschiede sind Frauen mit ihrer Arbeit oder ihrem Leben meist nicht weniger zufrieden als Männer. Zugleich spielt die gesellschaftliche Zustimmung zur Gleichberechtigung eine große Rolle: Die sozialen Normen sind hinsichtlich der gleichen Bezahlung von Männern und Frauen der wichtigste Erklärungsfaktor für die Einkommensungleichheit zwischen den Geschlechtern (Lalive und Stutzer, 2010).

## Chancen und Risiken der Corona-Maßnahmen

Die Pandemie hat durch die ergriffenen Interventionen (insbesondere Social Distancing, Lockdown, Homeoffice/Remote Work) in bisher nicht bekannter Weise in die gewohnten Arbeitsmuster und das Familienleben eingegriffen. In Bezug auf die Geschlechtergerechtigkeit birgt dies neben Risiken auch Chancen. Die eben ausgeführten Gründe stehen dabei nicht losgelöst nebeneinander, sondern greifen ineinander. Die durch die Maßnahmen disruptiv beschleunigte Digitalisierung der Arbeitswelt, die stärker als je zuvor ortsungebundenes und zeitlich flexibles Arbeiten in einer Vielzahl von Bereichen möglich macht, kann zu einem Wandel der sozialen Normen und den damit verbundenen Rollenmodellen innerhalb der Familien beitragen und so mittelbar die Anforderungen an Führungspositionen verändern. Zugleich beschleunigt sie den Prozess veränderter Kompetenz- und Qualifikationsanforderungen.

Lagen die Beschäftigtenanteile mit Homeoffice vor der Krise bei 10 % (Männer) bzw. 12 % (Frauen), stiegen sie in der Krise auf 26 % (Männer) bzw. 30 % (Frauen) (Demmelhuber et al., 2020). Vergleichbar, aber mit einem höheren Anteil bei Männern, waren die Werte für Eltern mit Kindern unter 12 Jahren (Bujard et al., 2020). 2018 waren Väter zu 14,6 % im Homeoffice tätig, während der Anteil bei Müttern nur 9,2 % betrug. Dieser Geschlechterunterschied ist dabei in Teilen Ausdruck der Tätigkeitsunterschiede, da Mütter häufiger in Berufen mit einem geringeren Homeoffice-Potenzial tätig waren/sind. Zugleich spiegeln sich auch die unterschiedlichen Anteile von Führungspositionen zwischen den Geschlechtern in den Zahlen wider: Bereits vor der Krise konnten 37 % der Beschäftigten im Bereich „Geschäftsführung und Vorstand" im Homeoffice arbeiten – ein klar männlich dominierter Bereich.

Im Lockdown hat die Mehrzahl der Unternehmen mit flexiblen Arbeitszeiten und Homeoffice auf die Schließungen von Kinderbetreuung und Bildungsangeboten reagieren können. Dies hat zu Veränderungen in der Familienarbeit geführt. Noch nie haben sich so viele Väter um ihre Kinder gekümmert: Lag die tägliche Stundenzahl für Haus- und Familienarbeit mit Kindern unter 16 Jahren bei Müttern vor der Krise bei 6,6 Stunden, hat sie sich während der Krise auf 7,9 Stunden erhöht. Väter waren vor der Pandemie im Durchschnitt mit 3,3 Stunden beteiligt. Sie haben ihre Zeit auf durchschnittlich 5,6 Stunden ausgeweitet (Bujard et al., 2020). Obgleich weiterhin Geschlechterunterschiede in der Sorgearbeit bestehen, hat sich die Differenz verringert.[2] Kritische Stimmen befürchten aber eine Retraditionalisierung, da Frauen von der Schließung der Kinderbetreuungsstätten und Bildungsstätten durch den einhergehenden Betreuungsaufwand stärker betroffen sind und häufiger ihre Arbeitszeit reduzieren müssen (z. B. Kohlrausch und Zucco, 2020). Der absolut höhere Zeitaufwand und nicht die Differenz zwischen den Geschlechtern ist ausschlaggebend.

Ob die beobachteten Veränderungen zu einem nachhaltigen Wandel der sozialen Normen führen werden, der überdies zu einer Verbesserung der Geschlechtergerechtigkeit beiträgt, ist noch unklar. Analysen vergleichbarer politischer Interventionen belegen, dass die Neuverteilung der Aufgaben im Haushalt anhaltende Auswirkungen auf die Geschlechterrollen und die Arbeitsteilung haben kann (Alon et al., 2020; Sevilla und Smith, 2020; Reichelt et al., 2020). Derartige Effekte könnten also auch durch die Corona-Maßnahmen ausgelöst werden. Obwohl Frauen in die Sorgearbeit stärker eingebunden sind und dies ihre Karriereoptionen kurzfristig beschränkt, eröffnet die Erfahrung einer stärkeren Beteiligung der Männer an der Sorgearbeit die Möglichkeit, dass diese auch zukünftig einen höheren Anteil als bisher übernehmen. Mittelfristig könnte dies die ungleiche Verteilung der Arbeit im Haushalt verringern und Normen und Rollenmodelle beeinflussen.[3]

Etwas gedämpft werden diese Erwartungen durch den Umstand, dass eine Ursache für den beobachteten höheren Sorgeanteil der Männer in der ungleichen Verteilung systemrelevanter Berufe lag. In Bereichen, in denen mehr Frauen tätig sind und die in der Krise als systemrelevant gelten (z. B. Einzelhandel, Kranken-/Altenpflege), mussten die Männer verstärkt die Kinderbetreuung übernehmen. Auch wenn sich die Anteile wohl wieder verringern werden, ist davon auszugehen, dass die Veränderungen (Homeoffice/Remote Work) auch nach der Krise nicht flächendeckend rückgängig gemacht werden. Dies unterstreichen auch entsprechend hohe Zustimmungswerte für das Homeoffice.[4] Die Erfahrung der Arbeitgeber:innen, dass die Leistungserbringung[5] ihrer Angestellten – zumindest näherungsweise – aufrechterhalten werden kann, wird zu einer höheren Akzeptanz von Homeoffice und digitalen Arbeitsmöglichkeiten auch nach der Krise beitragen.

Hierzu dürfte besonders die positive Einstellung gegenüber mobilem Arbeiten, flexiblen Arbeitszeitmodellen und Teilzeitarbeit bei Führungskräften beitragen. Ausschlaggebend für die Führungskräfte sind die flexible Ausgestaltung des Arbeitsalltags, die bessere Vereinbarkeit von Familie und Beruf; diese Aspekte werden durch positive Einschätzungen im Hinblick auf ein selbstständiges Arbeiten, zusätzliche Freiräume für lange verschobene Arbeiten sowie die Atmosphäre zu Hause ergänzt (Initiative Chefsache, 2020a). Viele Unternehmen erfuhren durch die Krise, dass mehr Homeoffice und mobiles Arbeiten ohne große Nachteile für den wirtschaftlichen Erfolg möglich sind. Dienstreisen konnten häufig durch Video- oder Telefonkonferenzen ersetzt werden und werden auch künftig an vielen Stellen entbehrlich bleiben. Die dadurch eingesparte Zeit erlaubt ein effizienteres Arbeiten bei größerer Vereinbarkeit von Beruf und Familie (vgl. Abbildung 2). Freiwerdende Mittel aus der Umstellung in den Unternehmen – auch durch mögliche Einsparpotenziale für Büroflächen – sollten verstärkt zur Verbesserung der Kinderbetreuungsangebote eingesetzt werden, um den eingeschlagenen Weg weiterzuverfolgen.

Allerdings gibt es – zumindest solange es zur Schließung der Bildungs- und Betreuungseinrichtungen für Kinder und Jugendliche kommt – auch Beschränkungen der neuen Flexibilität. Bei problematischer Kinderbetreuungssituation sind Arbeitsqualität und -quantität nicht aufrechtzuerhalten. So berichteten Befragte von Verschlechterungen von 50 % bei der Arbeitszeit, von 67 % beim Output und von 46 % bei der Qualität der Arbeit (Demmelhuber

2 Die Veränderungen in den Anteilen der Sorgearbeit werden durch weitere Befragungen gestützt: 43 % der Befragten gaben an, dass sich die Kinderbetreuung durch die Krise verändert hat. Mehr als die Hälfte sah zudem eine Angleichung der Anteile: 51 % teilen sich die Verantwortung in gleichem Maßen, bei 22 % übernehmen die Männer die Hauptverantwortung, bei 27 % haben die Frauen die Hauptverantwortung (Initiative Chefsache, 2020b).

3 In diese Richtung deuten auch die Ergebnisse einer Befragung zu den Arbeitszeitwünschen. Bei Müttern sind die Anteile deutlich gestiegen, die über flexiblere Arbeitszeiten (mehr als 40 %) oder eine Teilzeittätigkeit (fast 50 %) nach der Krise nachdenken. Wichtiger noch sind die Anstiege bei den Vätern: 35 % präferieren Teilzeit und 33 % eine flexiblere Arbeitszeit (Initiative Chefsache, 2020a).

4 Nach einer Befragung der Bertelsmann-Stiftung im April 2020 gehen mehr als 80 % der Befragten von einem verstärkten Fortbestand von Homeoffice und virtuellen Konferenzen nach der Corona-Pandemie aus. Von der Mehrheit werden zudem langfristige Änderungen des Arbeitslebens hin zu räumlich flexibleren Arbeitsmodellen (88 %) und einem zeitlich flexibleren Arbeitsleben (72 %) erwartet (Bertelsmann Stiftung, 2020).

5 Die Befragung von Demmelhuber et al. (2020) macht deutlich, dass die Arbeit im Homeoffice sowohl mit einer Verschlechterung als auch einer Verbesserung in Bezug auf Qualität und Quantität verbunden sein kann, beide Veränderungen sich aber in etwa die Waage halten.

Abbildung 2

**Erwartete Folgen der COVID-19-Pandemie für Frauen in Führungspositionen**

**Ergebnisse der Arbeitsmarkteingriffe** → **(Mögliche) Implikationen für Frauen in Führungspositionen**

Ergebnisse der Arbeitsmarkteingriffe:

- Angleichung der Zeiteinteilung für Beruf und Familie (insbesondere Kinderbetreuung) zwischen den Geschlechtern
- Neujustierung der Arbeitsorganisation (flexiblere Arbeitszeit, ortsunabhängige Arbeitsmodelle, weniger Geschäftsreisen)
- Erhöhte Akzeptanz und positive Einstellung der Führungskräfte zur veränderten Arbeitsorganisation
- Geringeres Streben nach Führungspositionen sowohl bei Männern als auch bei Frauen durch die Krise

(Mögliche) Implikationen für Frauen in Führungspositionen:

- Änderung der Einstellung gegenüber tradierten Geschlechterrollen durch initiierten längerfristigen Wertewandel
- Veränderung der geschlechterspezifischen Arbeitsteilung im Haushalt
- Verbesserte Vereinbarkeit von Familie, Karriere und Beruf für beide Geschlechter
- Verstärkte Nutzung freiwerdender Mittel (wegen Reisen, Büroflächennutzung) aus der Umstellung in den Unternehmen zur Verbesserung der Kinderbetreuung

Quellen: eigene Darstellung.

et al., 2020). Es ist davon auszugehen, dass diese drastischen Ergebnisse speziell auf die komplette (aber vorübergehende) Schließung der Bildungs- und Betreuungseinrichtungen zurückzuführen sind und sich daher nicht vollständig auf die Produktivität im Homeoffice nach der Krise übertragen lassen.

Die Corona-Maßnahmen werden langfristig in jedem Fall zu einer Neujustierung der Arbeitsorganisation führen (vgl. Mönnig et al., 2020; Alon et al., 2020; Sevilla und Smith, 2020). Die Veränderungen hin zu flexibleren Arbeitszeiten, ortsunabhängigen Arbeitsmodellen und weniger Geschäftsreisen können dazu beitragen, dass eine bessere Vereinbarkeit von Beruf, Karriere und Familie für beide Geschlechter erreicht wird. So entlasten die Maßnahmen die in der Sorgearbeit besonders engagierten Mütter nicht allein durch Veränderungen ihres Arbeitsplatzes, sondern erhöhen in gleicher Weise die Flexibilität für Väter und erlauben eine höhere Beteiligung an Kinderbetreuung und Haushaltsführung. Dass sich in dieser Hinsicht ein Wertewandel vollzieht, bestätigen aktuelle Befragungsergebnisse. War zu Beginn der Pandemie im April 2020 nur für 35 % der Männer eine Vereinbarkeit von Familie und Beruf durch Homeoffice wichtig, lag derselbe Anteil im September 2020 bereits bei über 50 % (Initiative Chefsache, 2020b). Dies kann zumindest als Indiz einer längerfristigen Bereitschaft der Männer gewertet werden, Familie und Beruf besser zu vereinbaren und damit die Rollenmodelle zu verändern. In diesem Zusammenhang könnte dann auch das Topsharing zunehmend an Bedeutung gewinnen, d. h. ein Modell des Aufteilens einer Führungsposition auf zwei Teilzeitkräfte. Dies wäre nicht nur für Frauen, sondern auch für Männer von Vorteil, da sie weniger Nachteile durch die Wahrnehmung familiärer Pflichten befürchten müssten (Schrenker und Zucco, 2020). Solche Entlastungen würden die Möglichkeit der Frauen und insbesondere Mütter zur eigenen Karriere verbessern. Frauen können daher langfristig am stärksten von den veränderten Bedingungen profitieren.

Die verbesserte Gleichberechtigung, der höhere Stellenwert der Vereinbarkeit von Familie und Beruf und die zusätzlichen Anforderungen, die orts- und zeitungebundenes Arbeiten an Führungskräfte stellen, haben aber auch eine Kehrseite, die die Anstrengungen zu mehr Frauen in Führungspositionen konterkarieren kann. Die veränderten Rahmenbedingungen beeinflussen nämlich gleichermaßen die Präferenzen. Äußerten im Januar 2020 (vor der Krise) 44 % der Frauen und 49 % der Männer den Wunsch in Führungspositionen aufzusteigen, sanken diese Anteile auf 11 % bei Frauen bzw. 21 % und dann 18 % bei Männern im April und September 2020 (Initiative Chefsache, 2020b). Im gleichen Zeitraum ist die Zufriedenheit mit der aktuellen Position unabhängig vom Geschlecht deutlich gestiegen.[6] Auch wenn diesen Zahlen eine hohe Volatilität innewohnt, könnten sie nachhaltige Verhaltensänderungen andeuten, wie ebenso bei der Veränderung der Arbeitsorganisation erwartet. Die Verringerung des Interesses an Führungspositionen ist dabei kritisch für die politischen und gesellschaftlichen Bemühungen einer Erhöhung der Frauenanteile.

## Implikationen

Obgleich die Förderung von Frauen in Führungspositionen bereits seit Langem gesellschaftlich und politisch diskutiert wird, hat sie durch die Gesetzgebung der EU-Kommission und die Arbeitsmarktveränderungen in der Corona-Krise eine praktische Beschleunigung erfahren. Die Maßnahmen habe dazu geführt, dass die Zeiteinteilung für Beruf und Familie, insbesondere für die Kinderbetreuung,

6 Zufriedenheit mit aktueller Position: Männer: Januar 24 %, September 45 %; Frauen: Januar 27 %, September 53 %.

zwischen den Geschlechtern ähnlicher geworden ist. Dies kann einen längerfristigen Wertewandel initiieren, aus dem sich Veränderungen der geschlechterspezifischen Arbeitsteilung im Haushalt ergeben. Hierzu haben die sprunghafte Digitalisierung von Arbeitsprozessen sowie die Ausstattung der Mitarbeiter:innen mit den entsprechenden Kommunikationstools beigetragen. Die Produktivität, die trotz aller Beschränkungen nicht vollständig rückläufig ist, unterstreicht dabei die Effektivität des Homeoffice.

Auch nach der Krise werden die flexiblen Arbeitsformen – insbesondere Homeoffice und weniger Geschäftsreisen – fortbestehen, wenn auch vermutlich in geringerem Umfang als gegenwärtig, und damit zu einer Änderung der Einstellung gegenüber den tradierten Geschlechterrollen führen. Trägt dies nachhaltig zu einer verbesserten Vereinbarkeit von Familie und Beruf bei, wird der zentrale Hindernisgrund auf bessere Karrierechancen für Frauen verringert. Hierzu tragen die Corona-Maßnahmen in klarer Weise bei. Kurzfristig können sich aufgrund der Einschränkung der Kinderbetreuung in der Pandemie Beeinträchtigungen in der Realisierung der Karriere (speziell der Aufstieg in Führungspositionen) für Frauen ergeben. Längerfristig wird die Akzeptanz mobiler Arbeitsmodelle sowohl für Frauen als auch für Männer zu einer besseren Vereinbarkeit von Familie und Beruf beitragen, wovon gerade die Frauen, auch im Hinblick auf die von der EU artikulierten Ziele, profitieren werden.

**Title:** ***Women as Leaders: Chances and Risks of the COVID-19 Pandemic***

**JEL Classification:** *J13, J16, J71*
